# Deciphering the Molecular Bittercode in Potato Protein Hydrolysates through a Sensoproteomics Approach

**DOI:** 10.1021/acs.jafc.5c10819

**Published:** 2025-12-26

**Authors:** Patrick T. Röhrl, Denise Ilogu, Colleen Demetriou, Oliver Frank, Verena K. Mittermeier-Kleßinger, Corinna Dawid

**Affiliations:** † Chair of Food Chemistry and Molecular Sensory Science, TUM School of Life Sciences, 9184 Technical University of Munich, Lise-Meitner-Str. 34, D-85354 Freising, Germany; ‡ TUMCREATE Ltd., Singapore 138602, Singapore; § Professorship for Chemosensory Food Systems, TUM School of Life Sciences, Technical University of Munich, Lise-Meitner-Str. 34, D-85354 Freising, Germany; ∥ 84503 Leibniz Institute for Food Systems-Biology at the Technical University of Munich, Lise-Meitner-Str. 34, D-85354 Freising, Germany

**Keywords:** peptides, sensoproteomics, potato, hydrolysate, protein, bitter, taste, Solanum tuberosum

## Abstract

Potato protein hydrolysates (*Solanum tuberosum* L.) exhibited a strong bitter off-taste. This study aimed to elucidate the origin of this bitter taste; therefore, literature-known bitter tastants such as amino acids, fatty acids, and fatty acid oxidation products were quantified. Using reconstitution experiments, the bitter taste of hydrolysates with low bitterness was explained by the amino acids l-leucine, l-histidine, l-lysine, l-arginine, l-tyrosine, l-isoleucine, l-phenylalanine, and l-tryptophan, as well as palmitic acid; however, the taste of the hydrolysates with high bitterness could not be fully elucidated utilizing literature-known tastants. Using a sensoproteomics approach, including activity-guided fractionation coupled with untargeted/targeted proteomics, 21 new bitter peptides were identified. These peptides showed bitter recognition thresholds ranging from 48 μmol/L (PAF) to 707 μmol/L (DDKDFLPF), suggesting that they are key contributors to the bitterness of the hydrolysate. Using these peptides, new UHPLC-MS/MS methods can be developed to guide the production of less bitter hydrolysates.

## Graphical abstract

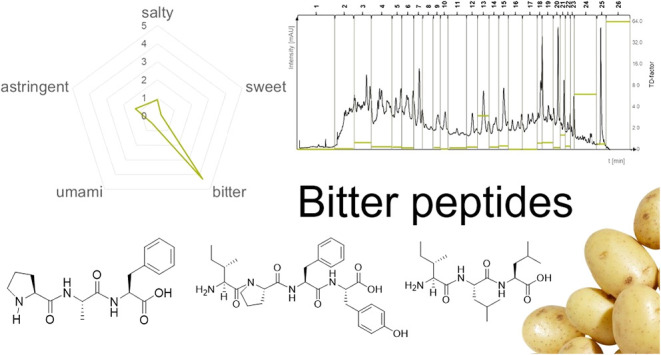

## Introduction

There is a rising demand for proteins to nourish the growing world population, which is projected to reach nine billion by 2050.^1^ Animal-based protein sources cannot sustain the population at the agricultural or environmental levels.^1^ Therefore, plant-based proteins are needed, especially proteins from new sources and side-stream products, such as the potato protein. During starch production, approximately 7 m^3^ of potato fruit juice is generated from one ton of potatoes.^2^ This potato fruit juice contains 20–25% protein (dry matter). Potato protein is produced through thermal precipitation of dissolved proteins.^2^ In Germany, the average potato starch production each year over the last 15 years has been 350,000 tons, which highlights the significant potential of this protein source.^3^

The potato protein mainly consists of protease inhibitors (50%), patatin (up to 40%), and other high-molecular-weight proteins (10%).^4^ Potato protein is one of the plant-based proteins with the highest percentage of essential amino acids in the total protein content, comparable to the amount in egg or casein protein.^5^ Furthermore, potato protein is deemed allergen-free, making it a useful alternative to legume-based proteins like soy or pea.^6^ Additionally, potato protein has favorable functional properties such as foam and emulsion stability, gel formation, and antioxidant activities.^6^ Conversely, potato protein, especially heat-coagulated potato protein, exhibits an unpleasant sandy texture.^7^ Moreover, potato protein exhibits poor solubility in water, with roughly 25–30% of proteins dissolved, which limits its application areas.^4^

To mitigate these problems, enzymatic or acidic hydrolysis can be applied to produce a more favorable and versatile protein.^4^ After hydrolysis, up to 80% of the potato protein is dissolved, which extends the corresponding application range.^4^ Moreover, the foam capacity and stability can increase up to 20-fold, and the oil-holding capacity can increase 5-fold.^2^ Furthermore, it has been shown that potato protein hydrolysates have even higher antioxidant activity than the protein itself and can be used to hinder the autoxidation of fatty acids.^4,8^ The nutritional values increase during hydrolysis because antinutritive protease inhibitors—e.g., from the potato inhibitor II (PI-2) and potato cysteine protease inhibitor (PCPI) families, which constitute 34% of the total protein—are digested into peptides and lose their inhibitory function.^4,9^ The disadvantage of the hydrolysis of proteins is the release of bitter peptides, which limits the consumer acceptance of protein hydrolysates. Studies on different products such as casein hydrolysate,^10,11^ whey protein hydrolysate,^12^ and cream cheese^13^ have shown that the bitter peptides generated upon hydrolysis are the reason for the bitter off-taste. Furthermore, the bitter taste of plant-based protein hydrolysates from chickpeas,^14^ soy,^15,16^ peas,^17^ canola,^17^ and wheat^18^ has been linked to peptides. However, no specific bitter peptides have been identified in publications focusing on these products or plant-based protein hydrolysates. Several studies have investigated the functional properties of potato protein hydrolysates,^2,4,8,19–25^ but no research has been conducted on the origin of their bitter taste. Research has, however, revealed the importance of fatty acids and fatty acid oxidation products in the bitter taste of potato fibers, which are another byproduct of starch production.^26^ These compounds could also be present in the hydrolysates and contribute to the bitter taste.

This study aims to apply the sensoproteomics approach to potato protein hydrolysates to identify key bitter peptides contributing to the bitter off-taste, so that in the future, the formation of bitter peptides can be avoided.^13^ The samples underwent activity-guided fractionation, as well as untargeted and targeted proteomics techniques to elucidate the bitter peptides. Furthermore, literature-known bitter compounds such as amino acids,^27^ fatty acids, and fatty acid oxidation products^28,29^ were quantified, and reconstitution experiments were conducted to evaluate their impact on the overall bitter taste.

## Materials and Methods

### Chemicals

The following chemicals were obtained commercially: acetonitrile (ACN), isopropyl alcohol, and ethanol (EtOH) (VWR Chemicals, Fontenay-sous-Bois, France); formic acid (FA) (Merk, Darmstadt, Germany); and (10*E*,12*E*)-9-hydroxy-octadecadienoic acid, (9*E*,11*E*)-13-hydroxy-octadecadienoic acid, 11,12,13-trihydroxy-octadec-9-enoic acid, 18-hydroxy-oleic acid (IS 2), 9,10,11-trihydroxy-octadec-1,2-enoic acid, 9,10,13-trihydroxy-octadec-11-enoic acid, and 9,12,13-trihydroxy-octadec-10-enoic acid (Larodan AB, Solna, Sweden). The l-amino acids, ammonium bicarbonate (NH_4_HCO_3_), ammonium acetate, deuterium oxide (D_2_O), methanol-d_4_ (MeOD), sodium deuteroxide (NaOD), (10*E*,12*Z*)-9-hydroxy-octadecadienoic acid, (9*Z*,11*E*)-13-hydroxy-octadecadienoic acid, ^13^C_18_linoleic acid, linolenic acid, linoleic acid, and palmitic acid were purchased from Sigma (Steinheim, Germany).

Water for ultrafiltration, solid-phase extraction (SPE), high-performance liquid chromatography (HPLC), and ultrahigh-performance liquid chromatography (UHPLC) was obtained from a Milli-Q Advantage A10 system (Millipore, Molsheim, France).

The peptides VDDDKDFLPF, DDKDFLPF, LVLPE, IPFY, ALL, ALI, AIL, AII, PAF, LTL, ITL, ITI, LTI, ELW, LLI, LIL, LII, III, IIL, ILL, and ILI were purchased from GenScript (Rijswijk, Netherlands), and the peptide LLL was obtained from peptides and elephants (Hennigsdorf, Germany).

The food-grade amino acids l-leucine, l-lysine, l-histidine, l-arginine, l-tyrosine, l-valine, l-isoleucine, l-phenylalanine, l-glutamine, l-glutamic acid, l-aspartic acid, l-methionine, l-threonine, glycine, l-serine, l-alanine, and l-proline were provided by Symrise (Holzminden, Germany). The amino acids l *-*tryptophan and l *-*asparagine monohydrate used for sensory analysis were purchased from Sigma (Steinheim, Germany). Bottled water (Evian, low mineralization: 405 mg/L) was used for the sensory analysis, and the pH was adjusted to 5.5 using formic acid. All chemicals used for sensory evaluation were obtained with purities >90%, which was verified utilizing quantitative nuclear magnetic resonance spectroscopy (qNMR).

Avebe provided the potato protein isolate (Veendam, Netherlands), and Solabia provided a potato protein hydrolysate (PANTIN Cedex, France). The remaining potato protein hydrolysates were purchased from Sigma (Steinheim, Germany) and Gerbu (Heidelberg, Germany).

#### Quantification of Basic Taste Compounds

##### Quantification of Amino Acids

A modified procedure was applied to quantify the free amino acids based on the stable isotope dilution analysis (SIDA).^27^ For the potato protein isolate and the hydrolysates, 990 μL of a 1 mg/mL solution was spiked with 10 μL of the internal standard solution containing the isotopically labeled amino acids (see Supporting Information Table S1). Every sample was analyzed in triplicate and measured on a 5500 QTRAP (Sciex, Darmstadt, Germany) equipped with an Acquity UPLC BEH Amide column (2.1 mm × 100 mm, 1.7 μm; Waters, 139 UK Ltd. Manchester, United Kingdom) using aqueous ammonium acetate (5 mmol/L) adjusted to pH 3.0 as solvent A and a mixture (95/5, v/v) of acetonitrile/aqueous ammonium acetate (5 mmol/L) adjusted to pH 3.0 as solvent B. Further details of the method are provided in the Supporting Information.

##### Quantification of Fatty Acids and Fatty Acid Oxidation Products

For the quantification of fatty acids and fatty acid oxidation products, a modified SIDA procedure was applied.^30^ All samples were quantified in triplicate. In the case of the potato protein isolate, 100 mg was suspended with 1 mL of methanol/water (50:50; v/v) and 25 μL of an internal standard mix (0.5 mM ^13^C_18_-linoleic acid and 0.5 mM 18-hydroxy oleic acid). The solution was extracted for 1 h at constant agitation (700 rpm) and then centrifuged for 10 min at 13.4 krpm. The supernatant was collected, and the residue was extracted twice again using 500 μL of methanol/water (50:50; v/v). The combined supernatants of all three extractions were membrane-filtered (0.45 μm) and analyzed using UHPLC-DMS-MS/MS. For the potato protein hydrolysates, 5 mg was combined with 2 mL of methanol/water (50:50, v/v) and 25 μL of the internal standard. The samples were extracted as described above, using only one extraction cycle due to the lack of any residue. All samples were measured on a 6500+ QTRAP mass spectrometer with a SelexION+ DMS cell (AB Sciex, Darmstadt, Germany) equipped with a Kinetex C18 column (150 mm × 10 mm, 1.7 μm; Phenomenex, Aschaffenburg, Germany) using aqueous ammonium acetate (5 mmol/L) adjusted to pH 5.0 as solvent A and a mixture (55:40:5, v/v/v) of acetonitrile, isopropanol, and aqueous ammonium acetate (5 mmol/L) adjusted to pH 5.0 as solvent B. Further details are provided in the Supporting Information.

### Ultrafiltration

The most (KH4) and least bitter potato protein hydrolysates (KH1) were subjected to ultrafiltration using a Sartoflow Smart system (Sartorius Stedim Biotech, Göttingen, Germany) equipped with a molecular weight cutoff (MWCO) filter membrane (Sartocon Slice 200 Hydrosart, cutoff 5 kDa, Sartorius Stedim Biotech, Göttingen, Germany) and ultrapure water at 4 °C. Ultrafiltration removed all high-molecular-weight (HMW) components. Thus, only low-molecular-weight (LMW) components remained. The LMW and HMW samples were lyophilized and stored at −20 °C, and solutions of 5 mg/mL were analyzed using UHPLC-ToF-MS for untargeted proteomics.

### Solid-Phase Extraction (SPE)

Solid-phase extraction (SPE) was utilized to separate highly polar compounds and salts from the LMW fractions of KH1 and KH4. The SPE cartridges (Chromabond C_18ec_, Macherey-Nagel, Duren, Germany) were conditioned with acetonitrile (ACN), 60/40 ACN/0.1% FA in H_2_O (v/v), and 0.1% formic acid (FA) in water. The sample was dissolved in 0.1% FA in water and added to the cartridges. After that, the sample was eluted three times with 0.1% FA in water (SPE1), once with 60/40 ACN/0.1% FA in H_2_O (v/v) (SPE2), and finally with 100% acetonitrile (SPE3). The solvent was removed (vacuum; 40 °C), and the samples were lyophilized twice and stored at −20 °C until further use. The SPE fractions (5 mg/mL) were analyzed using UHPLC-ToF-MS.

### Separation of the Fraction KH4_LMW_SPE2 Using Preparative High-Performance Liquid Chromatography (HPLC)

For the fractionation of KH4_LMW_SPE2, the fraction was dissolved in 0.1% FA in water (33 mg/mL), and 500 μL of the dissolved fraction was injected onto a Gemini C18 column (250 mm × 10 mm, 5 μm, 110 Å; Phenomenex, Aschaffenburg, Germany) with a guard column of the same type. The sample was separated using a flow rate of 4.7 mL/min and 0.1% FA in water as solvent A and acetonitrile as solvent B with the following gradient: 0 min 0% B, 3 min 6% B, 30 min 11% B, 39 min 15% B, 55 min 33% B, 57 min 100% B, 59 min 100% B, and 62 min 0% B. A total of 26 fractions were collected according to the UV signal (204 nm); the solvent was removed (vacuum, 40 °C), and the samples were lyophilized twice. The residues were used for taste dilution analysis (TDA) and untargeted proteomics via UHPLC-ToF-MS. The HPLC system (Jasco, Groß-Umstadt, Germany) comprised a binary pump system AS-2055 Plus and a Rh 7725i type Rheodyne injection valve (Rheodyne, Bensheim, Germany). An MD-2010 Plus diode array detector (Jasco, Groß-Umstadt, Germany), working in the 200–500 nm range, was used to monitor the effluent at 204 nm. The Chrompass Chromatography Data System (Jasco, Groß-Umstadt, Germany), version 1.10.0.5590, was used for data acquisition.

### Sensory Experiments

#### General Conditions: Panel Training

Fourteen panelists, who had no history of known taste disorders and had given informed consent to participate in the present sensory tests, were trained to become familiar with the taste language and methodologies using solutions of purified reference compounds in weekly training sessions for at least two years.^31^ Sensory analyses were performed in a sensory panel room at 22–25 °C using nose clips to prevent cross-modal interactions with olfactory cues. Before sensory analysis, the isolated fractions and compounds were freeze-dried twice and confirmed to be free of solvent traces according to qNMR measurements. The sensory analyses were performed using the sip-and-spit method, meaning the test solutions were not swallowed but expectorated.^32^

#### Taste Profile Analysis

Potato protein isolate (KPI) was suspended in water under constant stirring (6 g/100 mL, pH 5.5) and offered to the trained panel.^29^ The panel was asked to evaluate the bitter, sweet, sour, umami, salty, and astringent taste qualities on a scale from 0 (not perceivable) to 5 (strongly detectable). For each taste intensity, the average value was calculated for all of the panelists.

#### Comparative Taste Profile Analysis

The potato protein hydrolysates (KH1 to KH4) were dissolved in water and constantly stirred (6 g/100 mL; pH 5.5) and then presented to the panelists, who had to rate the sample’s taste qualities from 0 (not perceivable) to 5 (strongly detectable) in comparison to the KPI.^29^ The ultrafiltration fractions of KH1 and KH4 were dissolved at natural concentrations in water and adjusted to a pH of 5.5, and the taste was compared to the corresponding KH1 or KH4 (6 g/100 mL, pH 5.5). Furthermore, the SPE2 fractions of KH1_LMW and KH4_LMW were dissolved in water in their natural concentration (pH 5.5) and compared to the corresponding KH1_LMW or KH4_LMW fraction in the natural concentration (pH 5.5). The average value of each taste intensity was calculated for the whole panel.

#### Taste Dilution Analysis (TDA)

The HPLC fractions F1 to F25 were dissolved in water (pH 5.5), and due to poor solubility, F26 was dissolved in water with 2% ethanol (pH 5.5) and sequentially diluted 1:2 (v/v) with water (pH 5.5) or 2% ethanol in water (pH 5.5; F26). The dilution series of each fraction was presented to the trained panel in order of increasing concentration and evaluated for taste using a triangle test with water (pH 5.5) or 2% ethanol in water (pH 5.5; F26). The dilution step, in which the sample and blank could only be differentiated, was defined as the taste dilution (TD) factor.^33^ For each fraction, the TD factor was calculated by using the average value.

#### Determination of Human Taste Thresholds

Triangular tests were conducted with increasing peptide concentrations to determine the concentration at which the peptides were detectable (taste threshold). For each peptide, a 1 mmol/L solution (pH 5.5) was diluted 1:2 with water (pH 5.5) until the panelists could not differentiate between the peptide solution and water.^34^ The individual taste threshold was determined by the geometric mean of the first missed and last correctly identified concentrations. The average of the whole panel was calculated to obtain the taste threshold concentration.

### Untargeted Proteomics

#### Ultrahigh-Performance Liquid Chromatography–Time-of-Flight–Mass Spectrometry (UHPLC–ToF–MS)

To identify potential bitter peptides, a TripleToF 6600 mass spectrometer (Sciex) coupled to a Shimadzu Nexera X2 system (Shimadzu, Kyoto, Japan) was used in positive ionization mode (ESI+), and MS^2^ data were acquired using information-dependent acquisition mode (IDA). The ion spray voltage was set to 5500 V, the source temperature to 550 °C, the nebulizing gas to 55 psi (air), the heating gas to 65 psi (N_2_), and the curtain gas to 35 psi (N_2_). A Kinetex C8 column (100 mm × 2.1 mm, 1.7 μm, 100 Å; Phenomenex, Aschaffenburg, Germany) was utilized for chromatographic separation. An aliquot (10 μL) of the potato protein hydrolysates and the LMW, SPE2 fraction, and fractions F13, F24, and F26 (5 mg/mL) were injected using 1% FA in acetonitrile as solvent B and 1% FA in water as solvent A, at a flow rate of 0.3 mL/min.

The following gradient was used for chromatography:^13^ 0 min 5% B, 0.5 min 5% B, 14 min 40% B, 15 min 100% B, 16 min 100% B, 17 min 5% B, and 20 min 5% B. The ToF–MS scan was acquired from *m*/*z* 100–1500 with an accumulation time of 250 ms, a collision energy of 10 V, and a declustering potential of 80 V. Product ion spectra were monitored in high-resolution mode up to a maximum of 15 most abundant candidate ions per cycle with a mass tolerance of 20 ppm. MS^2^ spectra were acquired from *m*/*z* 100–1000 with an accumulation time of 50 ms. The collision energy (CE) was set to 35 V with a collision energy spread (CES) of 15 V, and the declustering potential (DP) was set to 80 V. The selection criteria for the precursor ion included intensities of >100 counts/s, exclusion after three occurrences, and an isotope window of 4 Da. Dynamic background subtraction was performed.

UHPLC–ToF–MS–IDA data were analyzed using the software MaxQuant (v.2.0.2.0; v.2.4.8.0).^35^ The default settings were used, and the digestion mode was set to “unspecific” because the enzyme used to produce the hydrolysates was unknown, and, often, a mixture of enzymes is used to generate protein hydrolysates.^20^ The minimum peptide length was set to 3, Sciex Q-TOF was selected as the instrument type, and a FASTA file for *Solanum tuberosum* (ID: 4113) was obtained from UniProt, containing only the reviewed proteins. For identification, the Andromeda score was used as a cutoff filter to lower the chance of false positives. At a score above 100, the number of false positives is very limited, but many less abundant peptides are discriminated. Hence, a second score cutoff set at >50 was used to identify the less abundant peptides.

### Targeted Proteomics

#### Ultrahigh-Performance Liquid Chromatography–Tandem Mass Spectrometry (UHPLC–MS/MS)

To verify the peptide sequences identified by means of untargeted UHPLC-TOF-MS in combination with database-driven identification, targeted LC-MS/MS experiments were performed on a 5500 QTrap LC–MS/MS system (Sciex, Darmstadt, Germany) connected to a Shimadzu Nexera X2 system (Shimadzu, Kyoto, Japan). The system was operated in the positive electrospray ionization mode, and the ion spray voltage was set at 5500 eV, the source temperature at 400 °C, the nebulizing gas at 0.38 MPa, the heating gas at 0.45 MPa, and the curtain gas at 0.24 MPa. For chromatographic separation, a Kinetex C8 column (100 mm × 2.1 mm, 1.7 μm; Phenomenex, Aschaffenburg, Germany), 0.1% FA in water as solvent A, and 0.1% FA in acetonitrile as solvent B were utilized. The following gradient was applied at a flow rate of 0.4 mL/min: 0 min 0% B, 15 min 40% B, 15.5 min 100% B, 17.50 min 100% B, 18 min 0% B, and 20 min 0% B. The column temperature was set to 40 °C, and 1 μL of the peptide solutions (5 μg/mL) or the LMW, SPE2, and HPLC fractions F13, F24, and F26 (5 mg/mL) were injected. Instrument control and data acquisition were performed using Sciex Analyst software (v 1.6.2).

#### In Silico Development of MRM Methods and Peptide Verification

To create multiple reaction monitoring (MRM) methods for the target peptides, the software Skyline (version 24.1.0.199) was used.^36^ To facilitate the selection of target transitions, Skyline libraries were built based on the untargeted proteomics data and *in silico* calculations using the included PROSIT prediction model.^37^ In the case of tri- and tetrapeptides, *a*-, *b*-, *c*-, *x*-, *y*-, and *z*-product ions were used for singly or doubly charged precursor ions. Only *b*- and *y*-product ions were considered for peptides with more than four amino acids. The samples were dissolved in 0.1% FA in water (1 mg/mL) and screened for *in silico* calculated mass transitions of the target peptides. The raw data was imported into Skyline for data evaluation. Only peptides with nonambiguous signals and five aligned mass transitions were deemed verified.^13^ In the case of tripeptides, four aligned mass transitions were deemed verified due to the smaller total number of possible mass transitions.

### Quantitative ^1^H NMR Spectroscopy

The synthesized reference peptides were dissolved in D_2_O (5–10.0 mmol/L), and NaOD was added depending on the solubility. An aliquot (600 μL) was transferred to 178 mm × 5 mm inner diameter NMR tubes (USC tubes, Bruker, Rheinstetten) and analyzed using a 400 MHz Avance III NMR spectrometer (Bruker, Rheinstetten, Germany) with the ERETIC 2 tool, applying the PULCON method. Instrument calibration and data processing were performed as detailed earlier by Frank et al. (2014).^38^ The specific proton resonance signal of the external standard l-tyrosine (4.08 mmol/L) at 7.10 ppm (d; 2H) was used for external calibration. Data evaluation was performed using software Topspin 3.6.0 (Bruker, Rheinstetten, Germany).

## Results and Discussion

Previous studies have reported increased bitterness in hydrolyzed plant-based proteins such as soy, pea, canola, chickpeas, and peanut hydrolysate compared to unhydrolyzed proteins.^14,15,17,39,40^ However, recent studies have not investigated the taste of potato protein hydrolysates; only studies concerning the functional properties of these hydrolysates have been published.^19^ Therefore, the taste profiles of one commercial potato protein isolate (KPI) and four hydrolysates (KH1–KH4) were investigated (Figure 1). The panelists were asked to evaluate the bitter, sweet, sour, umami, salty, and astringent taste qualities on a scale of 0 (not perceivable) to 5 (strongly detectable). Taste profile analysis indicated a significant increase in bitterness (α < 0.05, ANOVA), from 1.9 for the isolate (KPI) to 4.3 for the most bitter hydrolysate (KH4).

**Figure 1:**
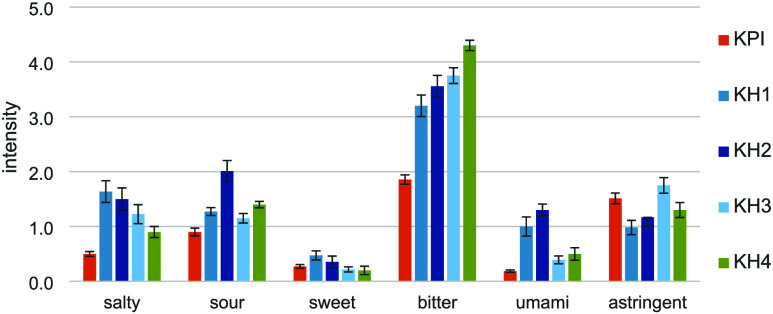
Taste intensities of the potato protein isolate (KPI) and four potato protein hydrolysates (KH1–KH4). The intensities of the individual taste qualities were rated on a scale of 0 (not perceivable) to 5 (strongly perceivable).

This observed increase in bitterness led to the conclusion that new bitter-tasting compounds were formed during the protein hydrolysis. Studies on peanut and soy protein hydrolysates have shown the release of bitter peptides upon hydrolysis.^39,40^ Even though their contribution to plant-based proteins has not been studied, it is known that taste-active peptides in protein-rich fermented foods like cream cheese are the origin of the bitter off-taste.^13^ Therefore, by applying the sensoproteomics approach, bitter peptides were expected to be identified with the help of activity-guided fractionation in combination with untargeted and targeted proteomics.

Moreover, research has indicated that the bitter compounds found in plant-based proteins and potato products, particularly certain fatty acids and their oxidation products, are key factors contributing to the bitterness perceived in pea protein and potato fibers.^26,29,30^ Furthermore, during hydrolysis, taste-active free amino acids are formed, which may contribute to the overall taste profile.^41,42^ Although the literature has provided quantitative data on the amount of free amino acids in hydrolyzed proteins, no reconstitution experiments have evaluated the effects of hydrolyzed proteins on taste. Therefore, free amino acids, fatty acids, and fatty acid oxidation products were quantified first to evaluate their impact on the taste of potato protein hydrolysates.

### Quantification of Basic Taste Compounds

During protein hydrolysis, peptides and free amino acids are generated.^39,42^ This increases the concentration of free bitter amino acids, which could be the cause of hydrolysates’ overall bitter off-taste. The amino acids l-leucine, l-lysine, l-histidine, l-arginine, l-tyrosine, l-valine, l-isoleucine, l-phenylalanine, and l-tryptophan are reported to taste bitter, with thresholds ranging from 4 to 80 mmol/L (Table 1).^43^ To investigate the impact of free amino acids on the taste profile, bitter, sweet, and umami-tasting amino acids were quantified in the potato protein isolate (KPI) and the most and least bitter hydrolysate (KH1, KH4) (Table 1). Aside from bitter amino acids, umami amino acids are especially interesting because umami-tasting compounds can mask bitterness and thus reduce the overall bitter taste.^44^ After quantification, the dose-overthreshold (DoT) factor was calculated to estimate the impact of the individual amino acids on the different taste qualities. The DoT factor is the ratio of the analyte’s concentration in a sample divided by its taste threshold concentration.^45^ If the DoT factor is above 1, the compound most likely contributes to the taste of the sample; if the DoT factor is between 1 and 0.1, synergistic effects could occur.^45^

**Table 1:** Taste Thresholds of Literature-Known Taste Active Compounds, Concentrations [μmol/kg] with Relative Standard Deviations (RSD [%]), and the Corresponding Dose over Threshold (DoT) Factors in Potato Protein Isolate (KPI) and the Least Bitter (KH1) and the Most Bitter Potato Protein Hydrolysates (KH4)^a^

		concentration [μmol/kg] ± RSD [%]^i^				DoT^j^	
tastant	threshold (μmol/L)	KPI	KH1	KH4	KPI	KH1	KH4
Bitter-Tasting Compounds							
l-leucine	11,000^c^	-	269,247.64 ± 4.3	275,757.26 ± 0.9	-	24.5	25.1
l-lysine	80,000^c^	302.76 ± 33.0	128,287.84 ± 2.5	83,327.03 ± 4.0	-	1.6	1.0
l-histidine	45,000^c^	-	45,549.06 ± 2.5	19,259.03 ± 8.3	-	1.0	0.4
l-arginine	75,000^c^	^b^	118,711.53 ± 3.0	80,451.26 ± 6.8	^b^	1.6	1.1
l-tyrosine	4,000^c^	174.57 ± 0.9	14,497.51 ± 3.6	38,361.52 ± 9.3	-	3.6	9.6
l-valine	20,000^c^	-	210.07 ± 1.3	32.18 ± 4.1	-	-	-
l-isoleucine	10,000^c^	-	319,371.64 ± 2.3	163,619.02 ± 8.0	-	31.9	16.4
l-phenylalanine	45,000^c^	-	122,450.35 ± 1.4	60,439.24 ± 5.3	-	2.7	1.3
l-tryptophan	4000^c^	-	30,384.51 ± 1.7	9,776.42 ± 5.0	-	7.6	2.4
linoleic acid	930^d^	33,946.84 ± 4.7	^b^	^b^	36.5	^b^	^b^
linolenic acid	280^d^	16,226.61 ± 0.3	^b^	^b^	58	^b^	^b^
palmitic acid	810^d^	46,401.87 ± 5.6	5,983.63 ± 9.0	6,681.76 ± 11.8	57.3	7.4	8.2
9-HODE (*trans*, *trans*)	350^d^	3493.04 ± 8.9	-	48.85 ± 12.0	10	-	0.1
13-HODE (*cis*, *trans*)	790^d^	3226.90 ± 28.6	-	-	4.1	-	-
13-HODE (*trans*, *trans*)	970^d^	25,900.95 ± 17.6	-	^b^	26.7	-	^b^
9-HODE (*cis*, *trans*)	790^d^	5896.32 ± 19.9	-	16.80 ± 20.2	7.5	-	-
11,12,13-THOA	130^e^	388.99 ± 18.8	74.09 ± 13.7	137.10 ± 8.1	3	0.6	1.1
9,12,13-THOA	130^e^	205.31 ± 19.1	^b^	^b^	1.6	^b^	^b^
9,10,11-THOA	130^e^	1,783.03 ± 5.7	576.72 ± 5.7	225.31 ± 1.7	13.7	4.4	1.7
9,10,13-THOA	80^e^	881.55 ± 11.8	195.63 ± 9.9	316.75 ± 4.7	11	2.4	4.0
Umami-Tasting Compounds							
l-glutamine	50,000^f^	115.90 ± 13.5	10,870.89 ± 28.4	-	-	0.2	-
l-asparagine	50,000^f^	730.22 ± 2.3	161,306.17 ± 5.7	84,223.03 ± 4.6	-	3.2	1.7
l-glutamic acid	1100^f^	-	79,701.18 ± 0.5	21,037.58 ± 11.6	-	72.5	19.1
l-aspartic acid	600^f^	-	92,968.45 ± 2.2	17,597.62 ± 8.0	-	154.9	29.3
Sweet-Tasting Compounds							
l-methionine	5000^g^	-	22,377.87 ± 2.4	28,166.91 ± 16.0	-	4.5	5.6
l-threonine	35,000^h^	-	167,153.92 ± 2.1	68,230.34 ± 7.8	-	4.8	1.9
glycine	25,000^h^	-	70,098.47 ± 0.8	61,925.05 ± 7.4	-	2.8	2.5
l-serine	25,000^h^	-	156,663.78 ± 5.0	67,136.29 ± 5.0	-	6.3	2.7
l-alanine	12,000^h^	-	148,560.65 ± 5.0	52,716.72 ± 5.0	-	12.4	4.4
l-proline	25,000^g^	-	16,252.71 ± 3.0	4687.15 ± 9.1	-	0.7	0.2

a DoT < 0.1 not shown; -: not detected.

b Under the limit of detection.

c Wieser et al., 1975 ^43^

d Lainer et al., 2019 ^28^

e Gläser, 2020 ^29^

f Toelstede et al., 2008b^46^

g Hufnagel et al., 2008 ^47^

h Wieser et al., 1977 ^48^

i Tastants were quantified in triplicate using UHPLC-MS/MS, and the mean value was determined.

j DoT factors were calculated as the quotient of the concentration [μmol/L] and the taste threshold concentration [μmol/L].

For KPI, no free amino acids were found with DoT factors exceeding 1. The bitter amino acids, l-isoleucine (KH1:31.9; KH4:16.4) and l-tryptophane (KH1:7.6; KH4:2.4), had at least twice as high DoT factors in KH1 compared to those in KH4, indicating a more substantial impact of these amino acids on the taste of KH1. The umami-tasting amino acids l-glutamic acid (KH1:72.5; KH4:19.1) and l-aspartic acid (KH1:154.9; KH4:29.3) also had at least three times higher DoT factors in KH1 than KH4, which correlates with the stronger umami taste of KH1 compared to that of KH4. Additionally, the sweet amino acids l-threonine (KH1:4.8; KH4:1.9), l-serine (KH1:6.3; KH4:2.7), and l-alanine (KH1:12.4; KH4:4.4) also showed higher DoT factors in KH1, comparable to those of the amino acids mentioned above. Overall, the DoT factors were higher in KH1 than in KH4, suggesting that the amino acids’ impact on the flavor profile of KH1 may be more pronounced.

Along with amino acids, fatty acids and fatty acid oxidation products have been described as the cause of the bitter taste in several plant-based proteins or products.^26,28–30^ Consequently, these were quantified in KPI, KH1, and KH4, and the DoT factors were calculated using the literature-known threshold concentrations (Table 1). Overall, a reduction in fatty acids and oxidation products was observed in the hydrolysates compared to the isolate, as fatty acids and oxidation products were either not detectable or present at concentrations below the limit of detection in the hydrolysates. In the hydrolysates, only the fatty acid palmitic acid had a DoT > 1 (KPI: 57.3; KH1:7.4; KH4:8.2), indicating its contribution to the hydrolysates’ bitterness. In addition, DoT factors >1 were determined for the fatty acid oxidation products 11,12,13-THOA (KPI: 3; KH1:0.6; KH4:1.1), 9,10,11-THOA (KPI: 13.7; KH1:4.4; KH4:1.7), and 9,10,13-THOA (KPI: 11; KH1:2.4; KH4:4.0). To verify the impact of these literature-known compounds on taste, taste reconstitution experiments were performed.

### Taste Reconstitution Experiments

Using the partial reconstitution experiments of the quantified basic taste compounds for KH1 and KH4, the impact of amino acids and fatty acids on the taste profile was assessed (Table 2). All amino acids with a DoT > 0.1 and/or palmitic acid were dissolved in Evian water at natural concentrations, and the pH was adjusted to 5.5.^29^ In one set of experiments, all amino acids and palmitic acid were included (pRec_AA + FA). In the second experiment, only amino acids (pRec_AA) were mixed to evaluate the amino acids’ contribution. The trained sensory panel evaluated the samples for salty, sour, sweet, bitter, umami, and astringent tastes on a scale of 0 (not perceivable) to 5 (strongly detectable; Table 2).

**Table 2:**
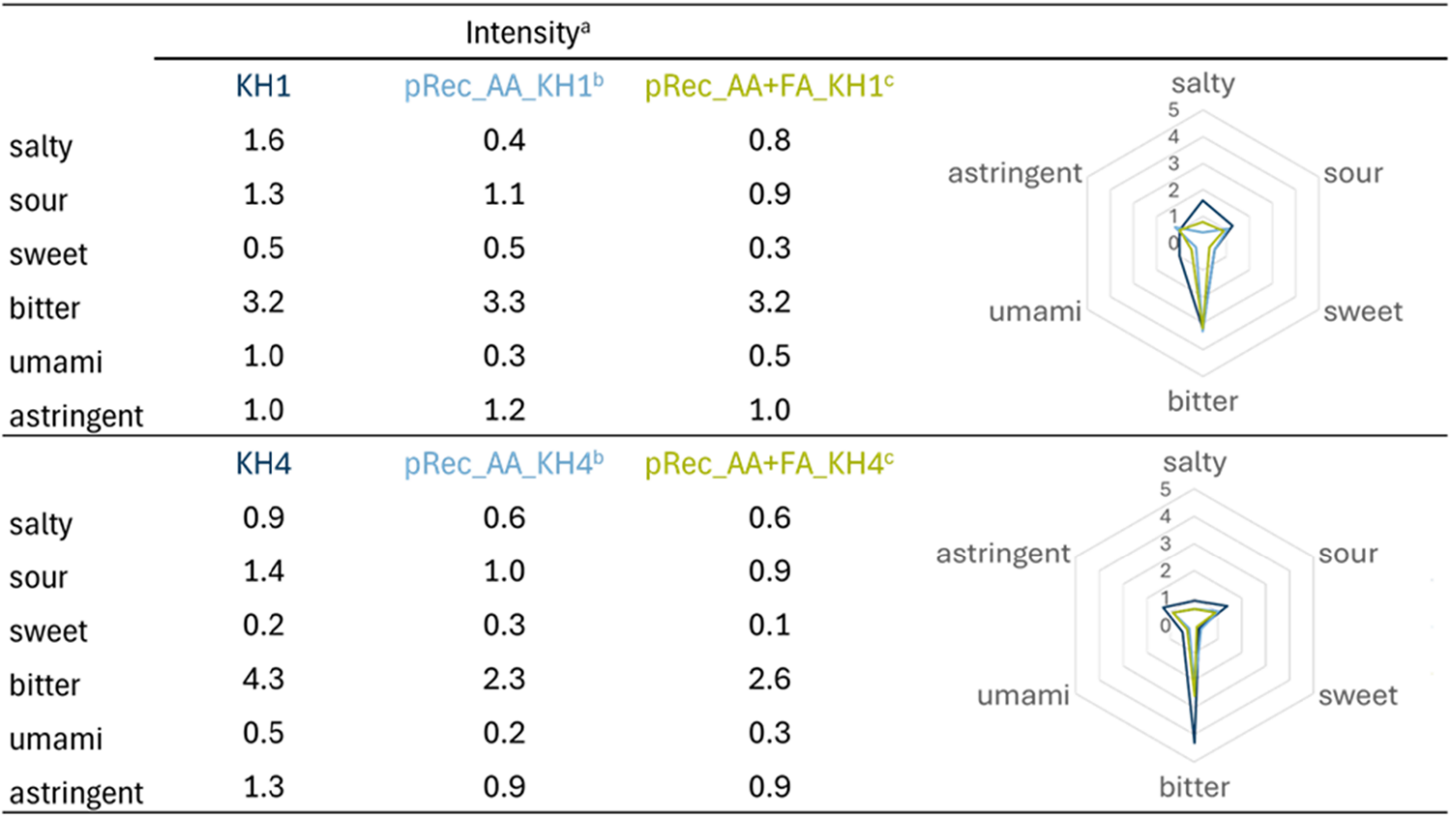
Taste Profile Analysis of the Least (KH1) and Most Bitter Potato Protein Hydrolysates (KH4) in Partial Reconstitution Experiments (pRec) Containing Amino Acids (AA) and Amino Acids/Fatty Acids (AA + FA)

a The intensities of the individual taste qualities were rated on a scale of 0 (not perceivable) to 5 (strongly perceivable).

b The partial reconstitution experiment (pRec_AA) contained the amino acids listed in Table 1.

c The partial reconstitution experiment (pRec_AA+FA) contained the amino acids and palmitic acid listed in Table 1.

In the case of the least bitter potato hydrolysate KH1, the taste qualities bitter (KH1:3.2; pRec_AA: 3.3; pRec_AA + FA: 3.2), sweet (KH1:0.5; pRec_AA: 0.5; pRec_AA + FA: 0.3), salty (KH1:1.6; pRec_AA: 0.4; pRec_AA + FA: 0.8), sour (KH1:1.3; pRec_AA: 1.1; pRec_AA + FA: 0.9), and astringent (KH1:1.0; pRec_AA: 1.2; pRec_AA + FA:1.0) were entirely explained by the amino acids l-leucine, l-lysine, l-histidine, l-arginine, l-tyrosine, l *-*isoleucine, l-phenylalanine, l *-*tryptophan, l-asparagine, l-glutamic acid, l-aspartic acid, l-methionine, l-threonine, glycine, l-serin, and l-alanine, and the fatty acid palmitic acid. The umami taste quality (KH1:1.0; pRec_AA: 0.3; pRec_AA + FA: 0.5) only partially matched those between KH1 and the reconstitution experiments. Other compounds, like umami-enhancing peptides, could be responsible for the stronger umami taste in the hydrolysate compared to the reconstitution experiments.^49^ However, in the current studies, nothing is reported about the umami peptides in potato protein hydrolysates.^49^

Like KH1, the taste of KH4 was predominantly influenced by amino acids and palmitic acid. The results from the reconstitution experiments indicated that the taste qualities of salty (KH4:0.9; pRec_AA: 0.6; pRec_AA + FA: 0.6), sour (KH4:1.4; pRec_AA: 1.0; pRec_AA + FA: 0.9), umami (KH4:0.5; pRec_AA: 0.2; pRec_AA + FA: 0.3), and astringent (KH4:1.3; pRec_AA: 0.9; pRec_AA + FA: 0.9) showed no significant differences (*p* < 0.05) when compared to the taste profile of KH4. However, there was a significant (*p* < 0.05) difference between the bitter taste of KH4 (4.3) and pRec_AA + FA (2.6). Thus, the bitter taste of KH4 was not explained by literature-known compounds, and other unknown compounds are needed to recreate the complete taste profile. Additionally, palmitic acid did not significantly affect the overall taste of the hydrolysate, as there was no substantial difference between pRec_AA and pRec_AA_FA in any taste quality (*p* < 0.05).

### Identification of Candidate Bitter Peptides Using the Sensoproteomics Approach

During hydrolysis, taste-active free amino acids are released. According to the reconstitution experiments, these contributed to the bitter taste of KH4, together with palmitic acid. However, the bitterness intensity of the hydrolysate did not fully match, indicating that additional bitter compounds, e.g., bitter peptides, were present.^14–18^ Recent studies have suggested that bitter peptides may cause the bitter taste described in chickpea, soy, pea, canola, and wheat gluten protein hydrolysates.^14–18^ To investigate the peptides responsible for the bitter taste of the potato protein hydrolysate, a sensoproteomics approach was applied.^13^ This approach combines the activity-guided fractionation and sensory techniques of the sensomics approach with untargeted and targeted proteomics techniques to facilitate the straightforward identification of taste-active or taste-modulating peptides. To implement this approach, both the least bitter (KH1) and most bitter hydrolysates (KH4) were subjected to fractionation.

#### Activity-Guided Fractionation

KH1 and KH4 were separated by molecular size utilizing a 5 kDa cutoff to yield a low- (LMW) and high-molecular-weight (HMW) fraction (Figure 2). The samples were lyophilized, and their taste (water, pH 5.5, and natural concentrations) was evaluated via comparative taste profile analysis (Supporting Information, Table S4). The LMW fractions of KH1 and KH4 were found to taste nearly as bitter as the corresponding hydrolysates (KH1:3.2; KH1_LMW: 2.9; KH4:4.3; KH4_LMW: 4.0). As a result, these LMW fractions were further fractionated using solid-phase extraction (SPE) to enrich the bitter peptides and remove highly water-soluble compounds such as amino acids. Therefore, the compounds were eluted three times with 0.1% FA in water (SPE1), followed by once with 60/40 ACN/0.1% FA in water (v/v) (SPE2), and once with 100% ACN (SPE3). After removing the solvent (vacuum, 40 °C) and lyophilizing twice, the fractions were analyzed using comparative taste profile analysis (Supporting Information, Table S4). Among the different SPE fractions, SPE2 of KH1 and KH4 (KH1_LMW_SPE2:2.7; KH4_LMW_SPE2:3.7) exhibited the highest bitter intensity, followed by SPE1 (KH1_LMW_SPE1:1.3; KH4_LMW_SPE1:1.0). In contrast, SPE3 only showed weak bitterness (KH1_LMW_SPE3:0.5; KH4_LMW_SPE3:0.7).

**Figure 2:**
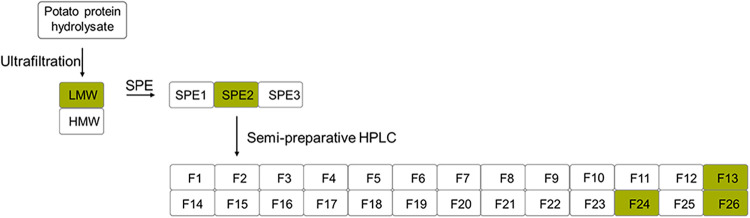
Fractionation scheme used to identify bitter-tasting compounds in the potato protein hydrolysate (bitter fractions highlighted in green). The hydrolysates (KH1 and KH4) were fractionated into high- (HMW) and low-molecular-weight (LMW) fractions using ultrafiltration, and the bitter-tasting LMW fraction was fractionated into three fractions using solid-phase extraction (SPE). The fraction SPE2 of KH4 was further fractionated into 26 fractions, F1–F26, using semipreparative HPLC.

For further fractionation, only the SPE2 fraction of the most bitter potato hydrolysate, KH4, was fractionated using semipreparative HPLC (Figure 3). Due to the complex chromatogram, several peaks were combined in each fraction, resulting in a total of 26 fractions (F1–F26). To focus on the fractions with the highest taste activity, taste dilution analysis (TDA) was performed. According to the TDA, the most bitter fractions were F26 (TD: 64), F24 (TD: 32), and F13 (TD: 16).

**Figure 3:**
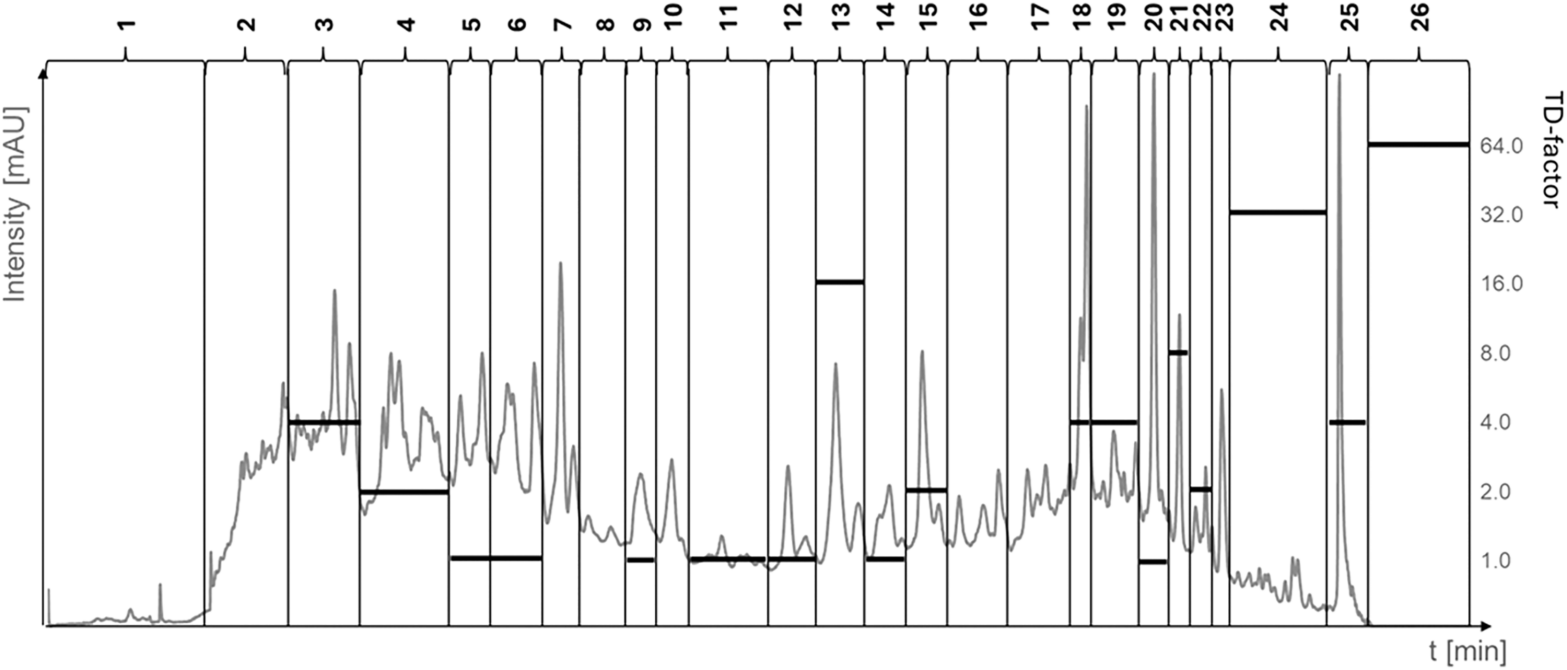
HPLC chromatogram of fractionated KH4_LMW_SPE2 with TD factors.

#### Untargeted Proteomics Using Ultrahigh-Performance Liquid Chromatography–Time-of-Flight–Mass Spectrometry and In Silico Peptide Sequencing

The LMW, SPE2 fractions, and HPLC fractions with the highest TD factors (F13, F24, and F26) were subjected to untargeted proteomics to elucidate the sequences of the target bitter peptides. The fractions were analyzed utilizing UHPLC-ToF-MS in information-dependent acquisition mode, and the obtained data were evaluated through database-driven identification using MaxQuant.^35^ To filter the peptides and evaluate whether a peptide sequence was a false or true positive hit, the Andromeda score was used, whereby a score of ≥100 indicates a slight possibility of being a false positive.^50^ Furthermore, as a lower-level filter criterion, an Andromeda score cutoff of 50 was chosen to detect less abundant peptides.

Applying these criteria, 79 and 96 peptides with scores of ≥50 were identified in KH1_LMW and KH4_LMW, respectively. Furthermore, 161 peptides with a score of ≥50 were identified in the SPE fraction KH1_LMW_SPE2. In the fraction KH4_LMW_SPE2, 139 peptides were identified, highlighting the need for HPLC fractionation to reduce the number of potential bitter peptides. Within the HPLC fractions of KH4, between seven (F24) and 38 (F13) peptides (scores >50) were detected. In addition, LVLPE was highly abundant in the most bitter fraction, F26. Due to its high Andromeda score (≥100), this peptide was included in the target list for further verification. To focus on peptides with the highest taste activity, the peptide sequences calculated *in silico* for the least bitter potato protein hydrolysate were compared. Overall, seven peptides were present in the LMW fractions of KH1 and KH4, and 29 were common in the SPE2 fractions of KH1 and KH4. Next, the fold change of the peptides, i.e., the ratio of their intensity in KH4 to KH1, was calculated. Only peptides with a fold change greater than three were included in the target peptide list, as these peptides were three times more abundant in the more bitter sample (KH4) and were thus most likely to contribute to the sample’s bitterness. This second filter criterion reduced the number of target peptides to 12.

#### Targeted Identification of Candidate Bitter Peptides

For the verification of the 12 target peptides obtained by determining the fold change and the additional peptide LVLPE identified by the highest intensity and a high Andromeda score in the most bitter fraction F26, the software Skyline^36^ was used to calculate the MRM transitions of each peptide *in silico*. Using these data, targeted UHPLC-MS/MS methods were developed with a maximum of 150 transitions per method. The hydrolysates and all fractions were screened on a 5500 QTRAP system by using the newly developed methods. To ensure unambiguous verification, only peptides with at least five aligned mass transitions and no further peaks were kept (Figure 4A). However, for the peptides ALL and LTL, two peaks with matching MRM patterns were visible (Figure 4B), and for LLL, three peaks with the same transition patterns were apparent (Figure 4C). These phenomena could be explained by the presence of leucine/isoleucine variants. Accordingly, four peptide sequences for peptides with two leucine residues (ALL and LTL) and eight peptide sequences for the peptide LLL were possible, thereby increasing the number of potential bitter-tasting peptides to 24. These 24 peptides were custom-synthesized (GeneScript, Netherlands; Peptides and Elephants, Germany), and the retention times and fragmentation patterns of the reference peptides and samples were compared, resulting in the verification of 22 peptides in KH4.

**Figure 4:**
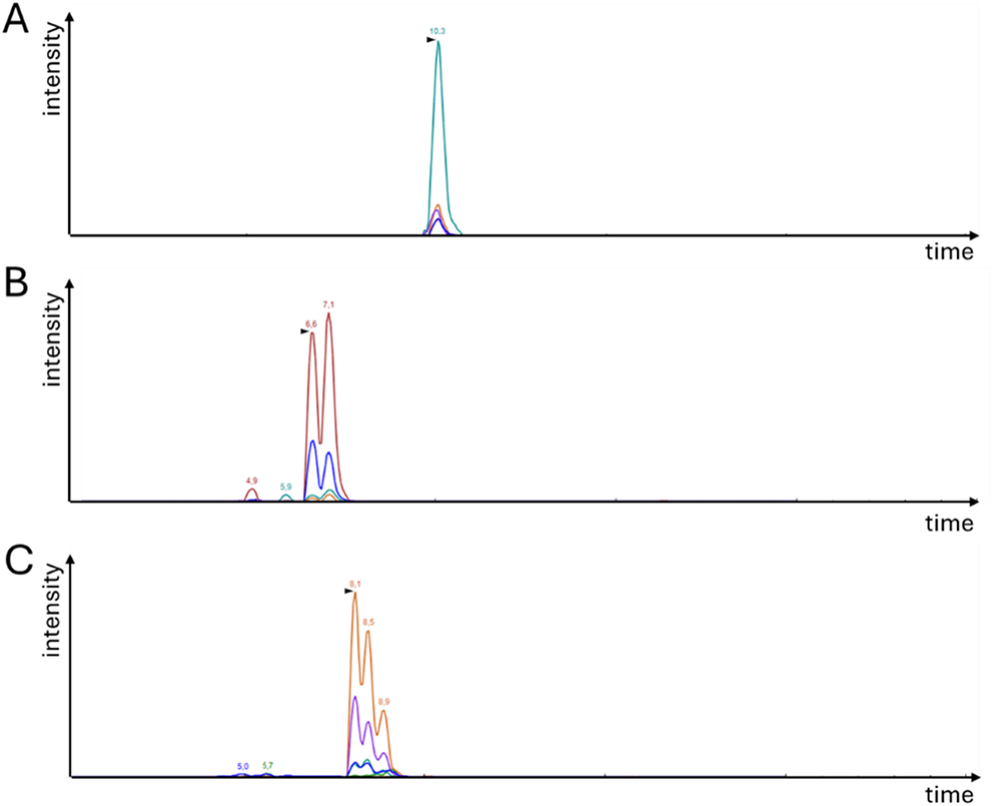
Mass transitions obtained for a peptide (A) without leucine (PAF), (B) with two leucine (ALL), and (C) with three leucine (LLL) residues.

### Sensory Activity of Bitter Peptides

After verification, the peptides’ taste activities were examined using human sensory experiments. The purity of the synthesized peptides was controlled before sensory analysis using UHPLC-ToF-MS and qNMR.^38^ To assess the intrinsic taste of the peptides, a 1 mM solution (pH 5.5) was evaluated at increasing concentrations using a triangular test (Table 3).^34^ The taste thresholds for bitterness ranged from 48 μmol/L (PAF) to 707 μmol/L (DDKDFLPF). Furthermore, different leucine/isoleucine isomers have different threshold concentrations. The AJJ peptides’ (J = I or L) threshold concentrations were in the range of 125.0 μmol/L (AIL) to 412.4 μmol/L (ALL), which represents a magnitude of more than 3-fold difference between AIL and ALL. Additionally, the JTJ peptides varied in their bitter thresholds, with a 4-fold difference between the lowest (88.4 μmol/L; LTL/ITL) and the highest (354.0 μmol/L; ITI). The same phenomenon was observed for the JJJ peptides.

**Table 3:** Bitter Threshold Concentrations [μmol/L] of the Newly Identified Peptides

peptide sequence	bitter threshold [μmol/L]
VDDDKDFLPF	108.0
DDKDFLPF	707.0
LVLPE	250.0
IPFY	63.0
ALL	412.4
ALI	353.6
AIL	125.0
AII	134.0
PAF	48.0
LTL	88.4
ITL	88.4
ITI	354.0
LTI	237.9
ELW	222.7
LLI	311.7
LIL	136.3
LII	295.3
III	176.8
IIL	176.8
ILL	94.7
ILI	292.7
LLL	200.5

In the literature, the taste of peptides containing only leucine with varying lengths, as well as the differences between d- and l-leucine for LL, have been previously evaluated.^51^ Ishibashi et al. highlighted that the bitter threshold decreases with length and found no difference between the d- and l-isomers. To the best of our knowledge, this is the first study to compare the taste threshold concentrations of leucine and isoleucine isomers of peptides.

Ney (1979) introduced the *Q*-value to predict the bitterness of peptides by calculating the sum of the hydrophobicity of each amino acid.^52^ His findings suggested that peptides containing isoleucine are more likely to taste bitter than those containing leucine. Although the isoleucine variants of peptides AJJ, JTJ, and JJJ are expected to be more hydrophobic and thus more bitter, this trend was not observed in this study. Overall, the threshold concentrations were similar to other bitter peptides reported in the literature, which range from 30 to 690 μmol/L.^13^ In summary, this study highlighted that amino acids released upon protein hydrolysis and palmitic acid substantially impact the taste of hydrolysates with a lower level of bitterness, as shown through reconstitution experiments for KH1. However, peptides released upon hydrolysis are important bitter tastants in potato protein hydrolysates and are necessary to explain the overall bitterness of hydrolysates with high bitter scores. Using a sensoproteomics approach, 22 bitter peptides were identified in the most bitter hydrolysate, exhibiting bitter thresholds between 48 and 707 μmol/L. To gain further insights into the concentrations of these peptides and their contribution to the overall bitterness of protein hydrolysates, new UHPLC-MS/MS methods need to be developed. This will simultaneously help navigate the production of (potato) protein hydrolysates toward pleasant-tasting products with low levels of bitter peptides and, thus, low overall bitterness.

## Supplementary Material



## Notes

Quantification of amino acids using UHPLC-MS/MS; concentration of the standards and internal standards used for the quantification of amino 53 acids (Table S1); MRM transitions used for the UHPLC-MS/MS quantitation of amino acids­(Table S1); quantification of fatty acids and oxidation products using UHPLC-DMS-MS/MS; MRM transitions used for the quantitation of fatty acids and fatty acid oxidation products 85 utilizing UHPLC–DMS–MS/MS (Table S3); comparative taste profile analysis of the ultrafiltration and SPE fractions (PDF)
