# Supplementary material for: Deciphering the Molecular Bittercode in Potato Protein Hydrolysates through a Sensoproteomics Approach
Source: J Agric Food Chem. 2025 Dec 26;74(1):1357–66. doi: 10.1021/acs.jafc.5c10819 (PMC12814519; doi:10.1021/acs.jafc.5c10819)
Supplement: Supplementary file 1 [file jf5c10819_si_001.pdf]

Supporting Information

Deciphering the Molecular Bittercode in Potato Protein Hydrolysate through a Sensoproteomics Approach

Patrick T. Röhrl[1,2], Denise Ilogu[1], Colleen Demetriou[1], Oliver Frank[1], Verena K. Mittermeier-Kleßinger[3], Corinna Dawid[1-4]

[1]Chair of Food Chemistry and Molecular Sensory Science, TUM School of Life Sciences, Technical University of Munich, Lise-Meitner-Str. 34, D-85354 Freising;
[2]TUMCREATE Ltd., Singapore, Singapore
[3]Professorship for Chemosensory Food Systems, TUM School of Life Sciences, Technical University of Munich, Lise-Meitner-Str. 34, D-85354 Freising
[4]Leibniz Institute for Food Systems-Biology at the Technical University of Munich, Lise-Meitner-Str. 34, D-85354 Freising

Corresponding author: corinna.dawid@tum.de; Tel.: 0049 8161 712901.

Quantification of amino acids using UHPLC-MS/MS.

The UHPLC-MS/MS measurements were performed at a flow rate of 0.4 mL/min on a Shimadzu Nexera X2 system consisting of a LC-30 AD pump, a SIL-30 AC autosampler, a CTO-30 A column oven and a CBM-20 interface (Shimadzu, Duisburg, Germany), with the following gradient: 0 min 90%B, 5 min 85%B, 8 min 55%B, 9 min 0%B, 11min 0%B, 12 min 90%B, and 14 min 90%B.

The system was operating in the positive electrospray ionization mode. The Ion spray voltage was set at 5500 eV, the source temperature was set at 500 °C, and nebulizing gas (0.38 MPa), heating gas (0.45 MPa), and nitrogen served as curtain gas (0.24 MPa) to effectively desolvate the ions. The system was run using the software Analyst 1.6.2 (Sciex, Darmstadt, Deutschland), and data evaluation was performed with the software Multiquant 3.03 (Sciex, Darmstadt, Deutschland).

For the internal standard solution, the isotopically labeled amino acids were dissolved in acetonitrile/water (10:90, v/v) (concentrations see Table S1). For calibration, a stock solution of all amino acids was prepared (concentrations listed in Table S1) in acetonitrile/ water (10:90, v/v). This stock solution was diluted 1:2, 1:4, 1:10, 1:20, 1:40, 1:100, 1:200, 1:400, 1:1000, 1:2000, 1:4000, 1:10000, and 1:20000. To each of these dilutions (990 µL), 10µL IS solution was added prior to UHPLC-MS/MS analysis. Response curves for the individual *L*-amino acids were created using linear regression, with the ratios of peak areas (the peak area of the analyte divided by the peak area of the internal standard) plotted against the ratios of concentrations (the concentration of the analyte divided by the concentration of the internal standard).

Table S1. Concentration of the standards and internal standards used for the quantification of amino acids.

amino acid standard	concentration [mg/L]
glycine	144.4
glycine-d_2	192.5
L-alanine	155.0
L-alanine-$^{13}C_3$	172.1
L-arginine	143.9
L-arginine-$^{13}C_6$	199.2
L-asparagine	142.2
L-asparagine-$^{15}N_2$	283.5
L-aspartic acid	145.5
L-aspartic acid-$^{13}C_4$, ^{15}N	172.1
L-glutamic acid	144.1
L-glutamic acid-$^{13}C_5$, ^{15}N	187.5
L-glutamine	192.1
L-glutamine-$^{13}C_5$	174.3
L-histidine	173.6
L-histidine-$^{13}C_6$	167.0
L-isoleucine	147.6
L-isoleucine-$^{13}C_6$	163.0
L-leucine	142.0
L-leucine-$^{13}C_2$	166.5
L-lysine	142.7
L-lysine-$^{13}C_6$, $^{15}N_2$	110.0
L-methionine	140.7
L-methionine-d_3	183.5
L-phenylalanine	178.4
L-phenylalanine-d_5	178.3
L-proline	145.4
L-proline-$^{13}C_5$, ^{15}N	199.8
L-serine	142.5
L-serine-$^{13}C_3$	178.6
L-threonine	141.3
L-threonine-$^{13}C_4$, ^{15}N	217.8
L-tryptophane	150.3
L-tryptophane-d_5	204.3
L-tyrosine	143.5
L-tyrosine-d_4	230.0
L-valine	144.3
L-valine-$^{13}C_5$, ^{15}N	156.9

Table S2. MRM transitions used for the UHPLC-MS/MS quantitation of amino acids; declustering potential (DP), entrance potential (EP), collision energy (CE), and collision cell exit potential (CXP).

compound	Q1	Q3	DP	EP	CE	CXP
glycine	76.0	30.0	51	10	17	14
glycine-d_2	78.0	32.0	51	10	17	16
L-alanine	89.9	44.1	1	10	13	8
L-alanine-$^{13}C_3$	93.0	46.0	41	10	15	8
L-arginine	175.1	70.0	71	10	31	10
L-arginine-$^{13}C_6$	181.0	73.9	61	10	29	10
L-asparagine	133.0	73.9	56	10	21	10
L-asparagine-$^{15}N_2$	135.0	75.0	66	10	21	12
L-aspartic acid	133.9	87.9	56	10	15	10
L-aspartic acid-$^{13}C_4$, ^{15}N	139.0	91.9	46	10	15	12
L-glutamic acid	148.0	83.9	41	10	23	12
L-glutamic acid-$^{13}C_5$, ^{15}N	154.0	88.1	61	10	23	12
L-glutamine	147.0	83.9	46	10	23	10
L-glutamine-$^{13}C_5$	152.0	88.0	46	10	25	12
L-histidine	156.1	110.1	66	10	19	8
L-histidine-$^{13}C_6$	162.0	115.0	61	10	21	8
L-isoleucine	132.0	86.0	126	10	15	10
L-isoleucine-$^{13}C_6$	138.1	91.0	51	10	15	12
L-leucine	132.1	86.0	116	10	15	10
L-leucine-$^{13}C_2$	134.0	87.0	51	10	15	10
L-lysine	147.1	84.0	56	10	23	10
L-lysine-$^{13}C_6$, $^{15}N_2$	155.1	90.0	51	10	23	12
L-methionine	150.0	103.9	71	10	15	12
L-methionine-d_3	153.0	107.0	51	10	15	12
L-phenylalanine	166.1	120.0	111	10	19	6
L-phenylalanine-d_5	171.1	125.1	86	10	21	6
L-proline	116.0	70.0	146	10	21	10
L-proline-$^{13}C_5$, ^{15}N	122.1	75.0	56	10	23	12
L-serine	106.0	59.9	51	10	15	8
L-serine-$^{13}C_3$	109.0	61.9	51	10	15	8
L-threonine	120.0	74.0	146	10	15	10
L-threonine-$^{13}C_4$, ^{15}N	125.0	79.0	136	10	27	12
L-tryptophane	205.1	188.0	51	10	15	10
L-tryptophane-d_5	210.1	192.1	46	10	15	8
L-tyrosine	182.0	136.0	46	10	19	6
L-tyrosine-d_4	186.0	140.1	61	10	19	8
L-valine	118.0	72.0	101	10	15	10
L-valine-$^{13}C_5$, ^{15}N	124.0	77.0	46	10	15	10

Quantification of fatty acids and oxidation products using UHPLC-DMS-MS/MS

The UHPLC-DMS-MS/MS measurements were performed at a flow rate of 0.35 mL/min on a Shimadzu Nexera X3 consisting of a LC-40D X3 pump, a SIL-40C X3 autosampler, a CTO-40-C column oven and a SCL-40 system controller (Shimadzu, Duisburg, Deutschland). The gradient for chromatographic separation was as follows: 0 min 15%B, 0.5 min 15%B, 2 min 30%B, 6 min 50%B, 17 min 71%B, 19 min 100%B, 21 min 100%B, 22 min 15%B, and 24 min 15%B.

The system was operating in the negative electrospray ionization mode. The ion spray voltage was set at -4500 eV, the source temperature was set at 450 °C, the DMS cell temperature was set at 225°C, the DMS offset to 3V, the separation voltage (SV) to 3500V, 1-propanol was used as modifier (363,6µL/min), and nebulizing gas (0.38 MPa), heating gas (0.45 MPa), and nitrogen served as curtain gas (0.24 MPa) to effectively desolvate the ions. System control was done using the software Analyst 1.6.3 (Sciex, Darmstadt, Deutschland), and data evaluation was performed with the software Multiquant 3.03 (Sciex, Darmstadt, Deutschland).

For the internal standard solution, $^{13}C_{18}$-linoleic acid (IS 1) and 18-hydroxy oleic acid (IS 2) were dissolved in methanol at a concentration of 0.5 mM. For the calibration, a stock solution of all the analytes was dissolved at a concentration of 0.2 mM in methanol, and diluted 1:2 until the concentration of 0.0004 mM to generate ten calibration points. To 1 mL of each point, 10.1 µL IS solution was added before the measurement. Linear regression was used for quantitation of fatty acids and fatty acid oxidation products by plotting the ratios of peak areas (the peak area of the analyte divided by the peak area of the internal standard) against the ratios of concentrations (the concentration of the analyte divided by the concentration of the internal standard).

Table S3. MRM transitions used for the quantitation of fatty acids and fatty acid oxidation products utilizing UHPLC–DMS–MS/MS; declustering potential (DP), entrance potential (EP), collision energy (CE), collision cell exit potential (CXP), and compensation voltage (CoV).

compound	Q1 [Da]	Q3 [Da]	DP [V]	EP [V]	CE [V]	CXP [V]	CoV [V]
palmitic acid	255.1	255	-120	-10	-16	-19	-19
linolenic acid	276.9	276.9	-110	-10	-10	-21	-20
linoleic acid	279.1	279	-175	-10	-14	-25	-18.5
(10*E*,12*E*)-9-hydroxy-octadecadienoic acid (9-HODE (*trans*, *trans*))	295.1	295	-55	-10	-12	-21	-16.5
(10*E*,12*Z*)-9-hydroxy-octadecadienoic acid (9-HODE (*cis*, *trans*))	295.0	295.2	-90	-10	-10	-23	-15.5
(9*E*,11*E*)-13-hydroxy-octadecadienoic acid (13-HODE (*trans*, *trans*))	295.0	295.1	-105	-10	-14	-27	-19.5
(9*Z*,11*E*)-13-hydroxy-octadecadienoic acid (13-HODE (*cis*, *trans*))	295.0	295.0	-40	-10	-8	-23	-17
9,10,11-trihydroxy-octadec-12-enoic acid (9, 10, 11-THOA)	329.0	329	-55	-10	-12	-29	1.5
9,10,13-trihydroxy-octadec-11-enoic acid (9, 10, 13-THOA)	329.0	329	-80	-10	-10	-31	-2
9,12,13-trihydroxy-octadec-10-enoic acid (9, 12, 13-THOA)	329.0	329.2	-65	-10	-12	-27	-7.5
11,12,13-trihydroxy-octadec-9-enoic acid (11, 12, 13-THOA)	329.0	329.2	-70	-10	-12	-27	-4
$^{13}C_{18}$-linoleic acid (IS 1)	297.1	297	-95	-10	-14	-25	-18.5
18-hydroxy oleic acid (IS 2)	297.2	297.2	-110	-10	-14	-19	-18

Table S4. Comparative taste profile analysis of the ultrafiltration and SPE fractions. *The intensities of the individual taste qualities were rated on a scale from 0 (not perceivable) to 5 (strongly perceivable).

	taste intensity*					
fraction	salty	sour	sweet	bitter	umami	astringent
KH1_LMW	1.9	1.1	0.4	2.9	1.0	0.9
KH1_HMW	0.2	0.2	0.3	0.6	0.2	0.6
KH1_LMW_SPE1	2.2	0.9	0.5	1.3	0.9	0.6
KH1_LMW_SPE2	0.2	0.3	0.1	2.7	0.1	1.0
KH1_LMW_SPE3	0.1	0.1	0.0	0.5	0.1	0.5
KH4_LMW	0.8	1.4	0.2	4.0	0.4	1.3
KH4_HMW	0.3	0.4	0.3	0.6	0.1	0.6
KH4_LMW_SPE1	1.3	1.3	0.4	1.0	0.4	0.4
KH4_LMW_SPE2	0.4	0.7	0.1	3.7	0.1	1.1
KH4_LMW_SPE3	0.2	0.2	0.1	0.7	0.0	0.9
